# Refractory IgG Warm Autoimmune Hemolytic Anemia Treated with Eculizumab: A Novel Application of Anticomplement Therapy

**DOI:** 10.1155/2016/9181698

**Published:** 2016-03-22

**Authors:** Kim Ma, Stephen Caplan

**Affiliations:** Department of Medicine, Division of Hematology, Jewish General Hospital, 3755 Chemin de la Côte-Sainte-Catherine, Room E-725, Montreal, QC, Canada H3T 1E2

## Abstract

Warm autoimmune hemolytic anemia (wAIHA) is the most common form of AIHA, with corticosteroids in first-line treatment resulting in a 60–80% response rate. Atypical wAIHA and IgG plus complement mediated disease have a higher treatment failure rate and higher recurrence rate. We report a case of severe wAIHA secondary to Waldenström macroglobulinemia with life threatening intravascular hemolysis refractory to prednisone, rituximab, splenectomy, and plasmapheresis. A four-week treatment of eculizumab in this heavily pretreated patient resulted in a sustained increase in hemoglobin and transfusion independence, suggesting a role for complement inhibition in refractory wAIHA.

## 1. Introduction

Warm autoimmune hemolytic anemia (wAIHA) constitutes about 75% of all AIHA [1]. The autoantibody involved in wAIHA is typically a polyclonal IgG immunoglobulin mediating removal of red blood cells (RBCs) extravascularly within the spleen, with rare cases involving IgA and IgM immunoglobulins.

Glucocorticoids are the mainstay of treatment for wAIHA with a response noted in about 80% of patients [2]. However, up to a third of patients will relapse. While there are no randomized clinical trials supporting second-line treatment, splenectomy and rituximab are considered effective second-line therapies after glucocorticosteroid failure [2].

Here, we report the efficacy of an off-label use of eculizumab (Soliris, Alexion Pharmaceuticals), a terminal complement inhibitor, in a case of refractory IgG and complement mediated wAIHA, in which there was clear activation of terminal components of the complement cascade.

## 2. Case Presentation

A 70-year-old man, known for Waldenström macroglobulinemia (WM) since 1997, has been in partial remission since 2002 with a stable IgM peak. He first developed IgG-mediated warm AIHA in 2007, which may develop in up to 10–20% of patients with WM [3]. He has since been treated with multiple courses of prednisone (2007, 2011, 2013, and 2014), rituximab (375 mg/m^2^ weekly for four weeks; 2011, 2013, and 2014), IVIG (2011), and splenectomy (June 2014) for multiple recurrences of wAIHA.

**Figure 1:**
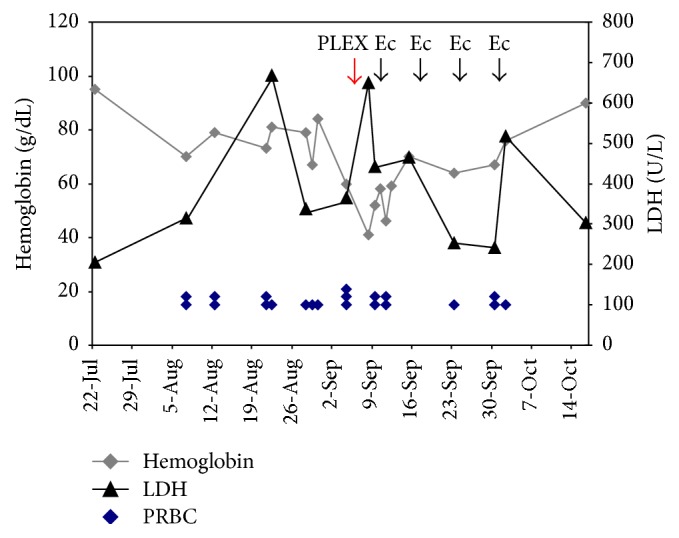
IgG-mediated warm autoimmune hemolytic anemia response to treatment. Hemoglobin and LDH are shown over the course of August to October 2014 including a hospital admission from September 8 to 12, 2014. Eculizumab was administered at a dose 600 mg IV. Each unit of PRBC is represented by a single point. LDH: lactate dehydrogenase; PRBC: packed red blood cells; PLEX: plasma exchange; and Ec: eculizumab.

In August 2014, the patient was started on his sixth course of prednisone (1 mg/kg) and fourth course of rituximab (375 mg/m^2^ weekly) for relapsed wAIHA. Despite three weeks of treatment, in September 2014, the patient developed life threatening anemia with severe hemoglobinuria and a hemoglobin nadir of 47 gm/L despite aggressive transfusion therapy (Figure 1). Laboratory workup revealed reticulocytosis (128 × 103), newly decreased haptoglobin (<0.10 g/L), and marked LDH. He was admitted to the hospital for urgent plasmapheresis. However, despite 1.0 volume exchange and six units of PRBC, the patient's hemoglobin remained critically low between 47 and 59 g/L.

A repeat direct Coombs test showed IgG and new C3d fixation on the surface of RBCs which had not been present previously. Allogeneic RBC antibody screen showed anti-E, anti-Fya, auto-anti-E, auto-anti-D, and nonspecific autowarm antibodies. Screen for cold agglutinins was negative.

In view of the patient's life threatening hemolytic anemia refractory to all conventional therapies, a trial of eculizumab 900 mg intravenously weekly was started based on the evidence of new complement mediated intravascular hemolysis. The patient felt rapid improvement in his fatigue consistent with similar rapid responses noted in PNH patients. Rapid symptomatic improvement following eculizumab treatment has been postulated to be due to correction of abnormal nitric oxide consumption [4]. He completed four weekly doses of eculizumab, and his hemoglobin stabilized at 90 g/L with a marked decrease in LDH (Figure 1) and no requirement for further transfusions.

He remained in stable partial remission until January of 2015, when he presented with relapse AIHA. Unfortunately, despite a second course of eculizumab, the patient passed away from refractory anemia.

## 3. Discussion

Eculizumab (Soliris, Alexion Pharmaceuticals), a terminal complement inhibitor, is a humanized monoclonal antibody that binds with high affinity to the human C5 complement protein and blocks the generation of proinflammatory C5a and C5b-9. It is currently FDA approved for the treatment of patients with PNH and atypical hemolytic uremic syndrome (aHUS) [5, 6].

Data on the use of eculizumab in AIHA is limited. There have been, to date, two case reports on the use of eculizumab in IgM-mediated cold agglutinin disease and one additional case report on the use of anticomplement therapy in IgM-mediated wAIHA [7–9]. Responses varied from 18 to 43 months, and duration of response varied from 1 to 36 months. A retrospective study by Barcellini et al. highlighted risk factors for severe refractory primary AIHA including mixed, atypical, and warm immunoglobulin G plus C mediated, as was the case in our patient [10]. More recently, Roth et al. reported a phase II study on the use of eculizumab in cold agglutinin disease (DECADE trial), which demonstrated that complement inhibition leads to a significant decrease in LDH and transfusion requirements [11]. Whether this is applicable to wAIHA has yet to be shown in a prospective trial.

Our case highlights a novel application of eculizumab in refractory wAIHA associated with complement mediated hemolysis in which terminal complement components, including C5, are involved in the pathogenesis of intravascular hemolysis. Although such cases are relatively rare, the severity and life threatening nature of the disorder demand novel therapeutic approaches. The observation of the dramatic response to eculizumab in this patient indicates a potential critical role of C5 inhibitors in this unique setting.

## References

[1] Bass G. F., Tuscano E. T., Tuscano J. M. (2014). Diagnosis and classification of autoimmune hemolytic anemia. *Autoimmunity Reviews*.

[2] Lechner K., Jäger U. (2010). How I treat autoimmune hemolytic anemias in adults. *Blood*.

[3] Bockorny B., Atienza J. A., Dasanu C. A. (2014). Autoimmune manifestations in patients with Waldenström macroglobulinemia. *Clinical Lymphoma, Myeloma and Leukemia*.

[4] Hill A., Rother R. P., Wang X. (2010). Effect of eculizumab on haemolysis-associated nitric oxide depletion, dyspnoea, and measures of pulmonary hypertension in patients with paroxysmal nocturnal haemoglobinuria. *British Journal of Haematology*.

[5] Varela J. C., Brodsky R. A. (2013). Paroxysmal nocturnal hemoglobinuria and the age of therapeutic complement inhibition. *Expert Review of Clinical Immunology*.

[6] Legendre C. M., Licht C., Muus P. (2013). Terminal complement inhibitor eculizumab in atypical hemolytic-uremic syndrome. *New England Journal of Medicine*.

[7] Röth A., Hüttmann A., Rother R. P., Dührsen U., Philipp T. (2009). Longterm efficacy of the complement inhibitor eculizumab in cold agglutinin disease. *Blood*.

[8] Gupta N., Wang E. S. (2014). Long-term response of refractory primary cold agglutinin disease to eculizumab therapy. *Annals of Hematology*.

[9] Chao M. P., Hong J., Kunder C., Lester L., Schrier S. L., Majeti R. (2015). Refractory warm IgM-mediated autoimmune hemolytic anemia associated with Churg-Strauss syndrome responsive to eculizumab and rituximab. *American Journal of Hematology*.

[10] Barcellini W., Fattizzo B., Zaninoni A. (2014). Clinical heterogeneity and predictors of outcome in primary autoimmune hemolytic anemia: a GIMEMA study of 308 patients. *Blood*.

[11] Roth A., Duhrsen U., Bommer M. Complement inhibtion with eculizumab in patients with cold agglutinin disease (CAD): results from a prospective phase II trial (DECADE trial).

